# Supplementary figures and images for: In vivo monitoring of leukemia-niche interactions in a zebrafish xenograft model
Source: PLoS One. 2024 Aug 30;19(8):e0309415. doi: 10.1371/journal.pone.0309415 (PMC11364250; doi:10.1371/journal.pone.0309415)

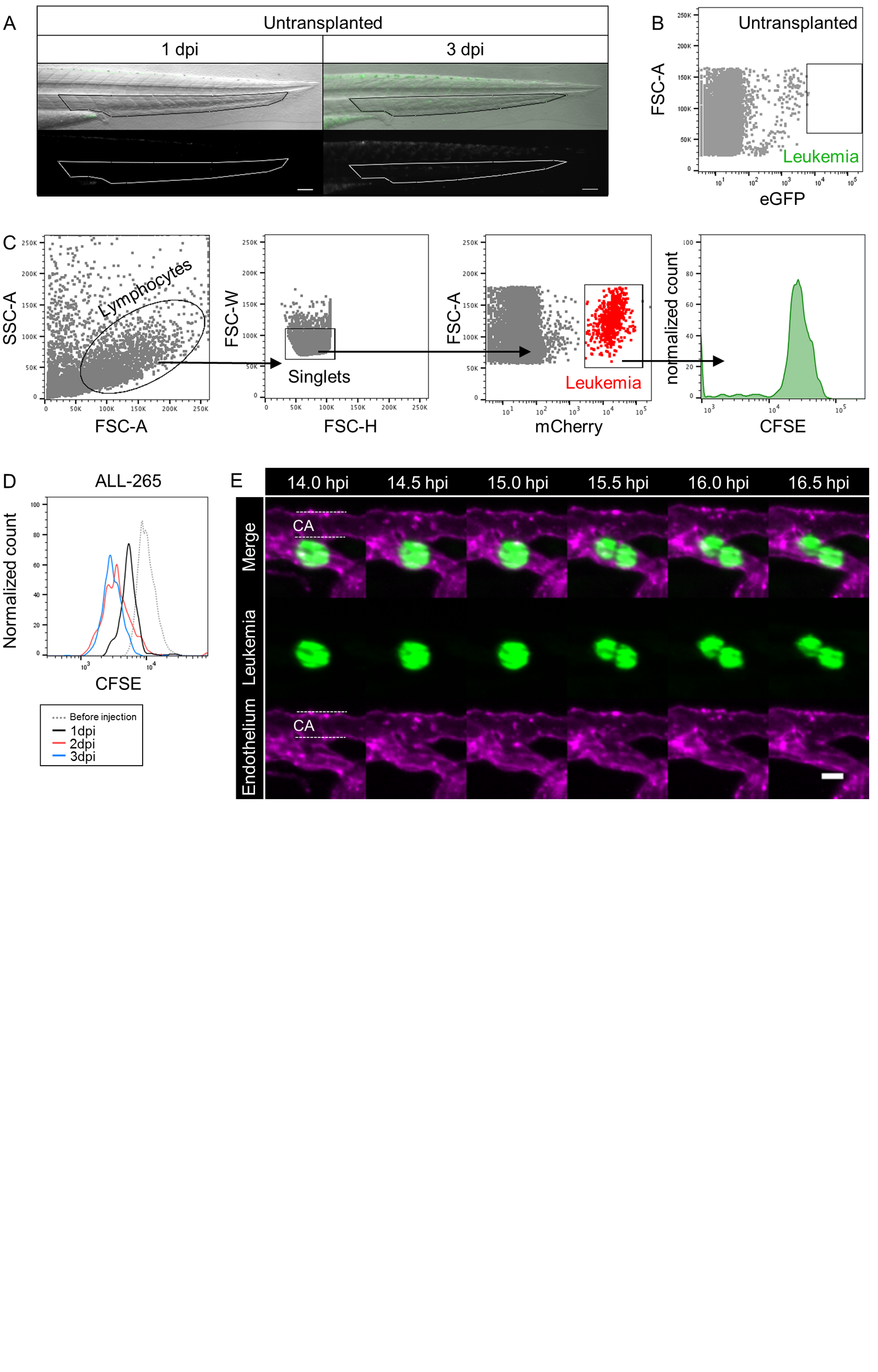

Supplement: S1 Fig — (A) Representative fluorescent (lower panel) and brightfield (merge, upper panel) images of the caudal part of an untransplanted larva at 1 and 3 dpi. The outline of the niche area is highlighted and was used to assess the CTCF of the larvae. (B) Dot plot of the flow cytometry measurement of an untransplanted sample. Grey dots show cells in singlets gate; green dots show NALM-6 cells. For detailed gating see S2A and S2B Fig. (C) Gating strategy for single cell suspension of transplanted larvae: ‘Lymphocyte’ gate was established using FSC-A/SSC-A of a pure leukemia sample (not shown). This gate was applied on the target sample and processed further. ‘Singlets’ were gated using FSC-H/FSC-W. mCherry positive ‘Leukemia’ cells (here NALM-6) were selected by gating on mCherry positive events using mCherry/FSC-A. These cells were used to analyze CFSE intensities and distribution patterns. (D) Representative histograms of the CFSE signal of PDX-265 at daily measurements of 10 pooled larvae. (E) Six frames of a time lapse video between 14 and 16.5 hpi with a frame interval of 30 min are depicted. Upper row shows the merged image of eGFP+ NALM-6 cells (green, middle row) and kdrl:mCherry endothelial cells (magenta, lower row). These images illustrate a NALM-6 cell located in a vessel for an extended period of time. Between 15 and 15.5 hpi the NALM-6 cell divided. CA: Caudal artery (outlined with white dashed lines). Scale bar: 10 μm. (TIF) [file pone.0309415.s001.tif]

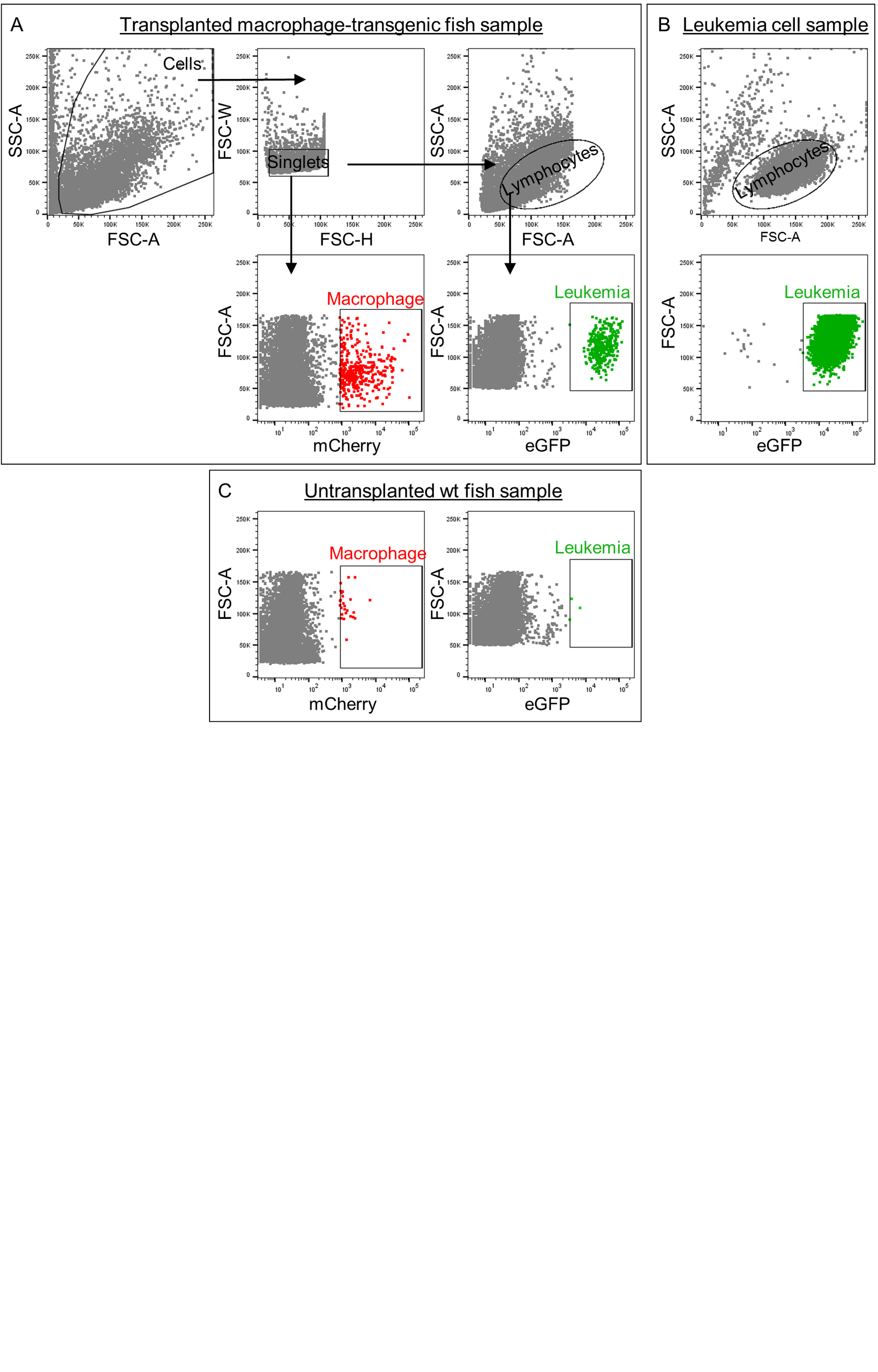

Supplement: S2 Fig — (A) ‘Cells’ were selected using FSC-A/SSC-A. ‘Singlets’ were gated using FSC-H/FSC-W. mCherry positive ‘macrophages’ were selected by gating on mCherry positive events using mCherry/FSC-A. ‘Leukemia cells’ were detected by applying the ‘lymphocytes gate that was drawn using a leukemia cell sample (see (B)) on the singlets gate (FSC-A/SSC-A). Next, events that were positive for the fluorescent marker (here: eGFP) were considered as leukemia cells by eGFP/FSC-A. (B) Exemplary gates of the pure LC sample. (C) Exemplary plots of untransplanted larvae sample. (TIF) [file pone.0309415.s002.tif]

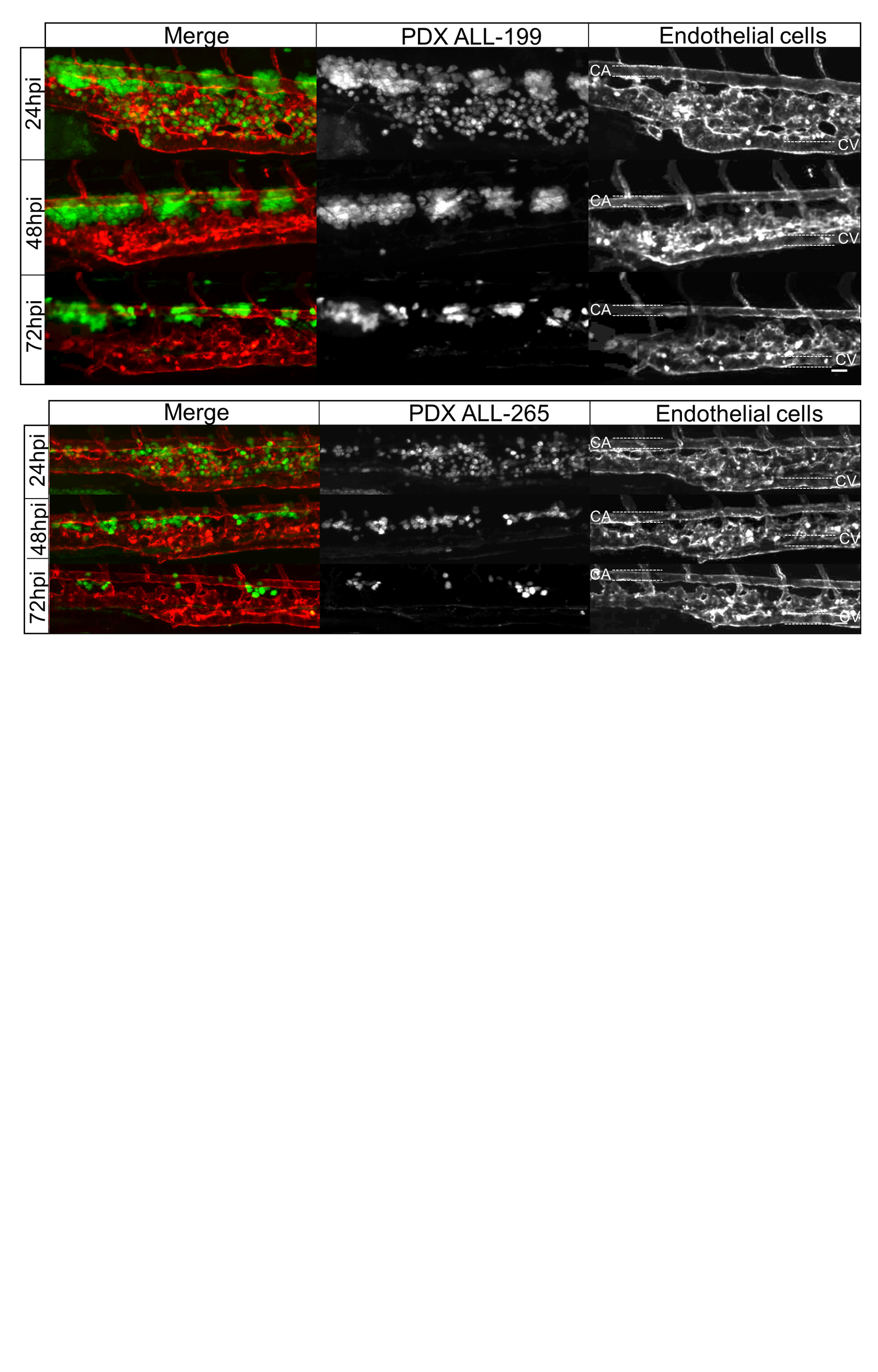

Supplement: S3 Fig — Confocal microscopy of the caudal parts of either one representative Tg(kdrl:mCherry) larva, transplanted with PDX-ALL-199 cells (top) or PDX-ALL-265 cells over the course of three days is depicted. Left panel shows merged images of LCs (green, middle panel) and the endothelium (kdrl:mCherry) (red, right panel). CV: Caudal vein (outlined with white dashed lines), CA: Caudal artery (outlined with white dashed lines). Scale bar 50 μm. (TIF) [file pone.0309415.s003.tif]

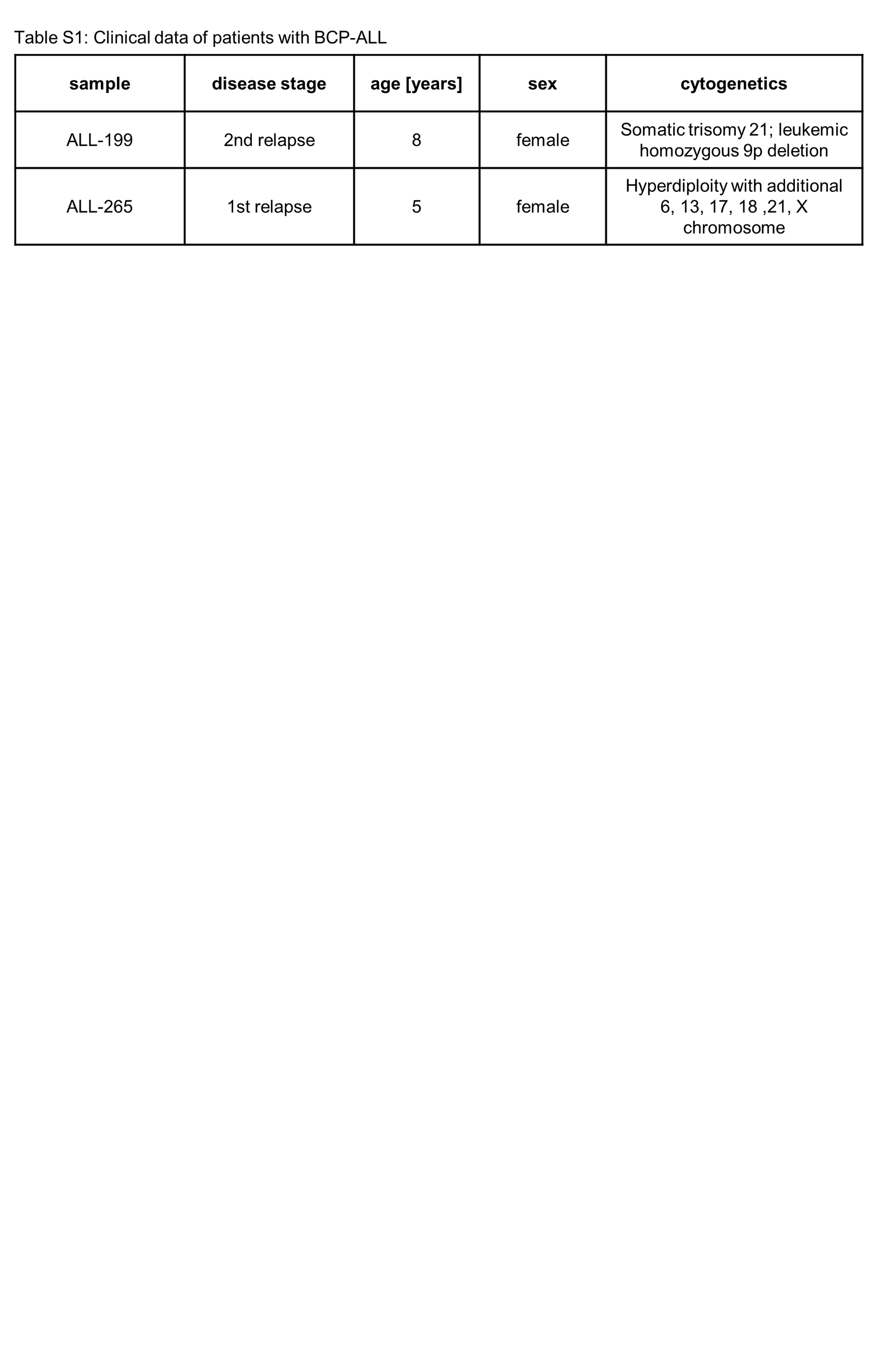

Supplement: S1 Table — (TIF) [file pone.0309415.s004.tif]
