# Supplementary material for: In vivo monitoring of leukemia-niche interactions in a zebrafish xenograft model
Source: PLoS One. 2024 Aug 30;19(8):e0309415. doi: 10.1371/journal.pone.0309415 (PMC11364250; doi:10.1371/journal.pone.0309415)
Supplement: Attachment — Submitted filename: Questions to authors.docx [file pone.0309415.s005.docx]

*In vivo* monitoring of leukaemia-niche interactions in a zebrafish xenograft model.

It is a very promising tool and I hope you will be able to investigate even more on this because it might provide, in next future, a concrete way to understand the interactions between cancer stem cells and their niche and on how both promotes the changes in the counterpart.

Briefly:

- Abstract: brief but straight to the point.
  - Line 30: comma after “transplant”.
- Introduction: well written but poor from the BCP-ALL side. I would like you to describe and widen the leukaemia part.
  - Line 55-58: the whole period is very complex and not well written, please re-write it.
- Mat and Methods: very well written. The authors have meticulously described all the tools, techniques, materials and reagents used in the study.
  - Line 96: please provide supplements of RPMI 1640 medium i.e. pen/strep, glutamine addition.
  - Line 125: comma after “)”.
  - Line 126: comma after “CHT”.
  - Line 129: change was with were determined.
- Results: well written even though some points must be cleared up.
  - Line 159-161: not easily comprehensible. Please, re-write the sentence.
  - Line 171: proliferate-**d**.
- Discussion: This part is well structured and substantiates authors' findings.
  - Line 345-347: re-write the sentence in a clearer way.

Considerations and questions to be answered (does not follow the order of the presented results):

Leukaemic Stem Cells (LSCs) reside within the leukaemic stem cell niche where their fate is decided. It is also well known that, within an heterogenous population, only a very small percentage comprehends leukaemic stem cells. In your latest part of the results, you show that almost the entirety of the injected LCs are capable to extravasate and colonize the niche. This should mean that you have a very rich stem population at time 0 of injection.

- Is it true?
- Do you have any idea why almost all of your cells extravasate between day 2 and 3 post injection and almost none remains in the vessel?
- Could you provide prof or idea of how NALM-6 cell line changes when it extravasates (i.e. expression of different adhesion markers for example, different metabolism)? It could be interesting to understand if, when extravasated, cells population enriches in terms of stemness.
- Have you gone further 3 days post injection to verify what happens to your cells in the CHT? Do you think it could be feasible? Do you think they might stay there or maybe colonize other parts of the animal?
- Could you describe a little bit further, in the intro section or in the discussion, the CHT part of the zebrafish and how it might interact and sustain ALL homing? You often speak about the niche but you never describe it very well.
- It could be useful for the reads if you indicate with a text the position of the vessels in the last images you presented.
